# *VDR* Gene Polymorphisms and Inter-Individual Variability in Response to Resistance Training

**DOI:** 10.3390/genes17020137

**Published:** 2026-01-27

**Authors:** Chen Yang, Yanchun Li

**Affiliations:** 1 College of Physical Education, Shihezi University, Shihezi 832003, China; ychen@shzu.edu.cn; 2 Department of Exercise Biochemistry, Exercise Science School, Beijing Sport University, Beijing 100084, China; 3 China Institute of Sport and Health Science, Beijing Sport University, Beijing 100084, China; 4 Beijing Key Laboratory of Sports Performance and Skill Assessment, Beijing 100084, China; 5 Key Laboratory for Performance Training & Recovery of General Administration of Sport, Beijing 100084, China

**Keywords:** *VDR*, gene polymorphisms, resistance training, muscle strength, bone density

## Abstract

**Background**: Vitamin D receptor (*VDR*) gene polymorphisms are linked to muscle and bone physiology, yet their influence on individual differences in resistance training adaptations, especially between sexes, is not well understood. **Methods**: In total, 191 healthy Chinese Han adults (94 men, 97 women) completed a 12-week, twice-weekly resistance training program (squat and bench press). Key indicators of strength, power, body composition, and muscle morphology were assessed before and after the intervention. Participants were genotyped for *VDR* polymorphisms (rs731236/*TaqI*, rs7975232/*ApaI*, rs1544410/*BsmI*, rs2228570/*FokI*). Data were analyzed to compare responses across genotype groups. **Results**: Training induced significant improvements in multiple outcomes. Overall, the AG genotype of rs731236 and the CT genotype of rs1544410 were associated with greater gains in bone mineral content. Sex-specific analyses revealed distinct patterns: in women, the rs731236-AA genotype correlated with better strength and power gains, while the AG genotype linked to greater body composition improvements. In men, the rs1544410-CC genotype was associated with superior lower-limb muscle growth. The rs7975232 showed no significant overall effect, and rs2228570 deviated from Hardy–Weinberg equilibrium. **Conclusions**: *VDR* gene polymorphisms, particularly rs731236 and rs1544410, are associated with inter-individual variability in resistance training responses among Chinese Han adults, demonstrating clear sex and phenotype specificity. These findings offer preliminary support for genotype-informed personalized training.

## 1. Introduction

Resistance training is a well-established intervention for enhancing muscle strength, inducing muscle hypertrophy, improving body composition, and increasing bone mineral density, conferring definitive benefits in the prevention of sarcopenia and osteoporosis [1,2]. A significant challenge, however, is the substantial inter-individual variability observed in response to identical training regimens. This heterogeneity is largely rooted in genetic factors [3]. Key exercise phenotypes—including maximal oxygen uptake, muscular strength, and body composition—demonstrate high heritability [3,4]. In exercise science, this differential adaptability is conceptually framed within the “gene-exercise interaction” paradigm, which explains how a standardized training stimulus elicits divergent adaptive outcomes based on an individual’s genetic makeup. Identifying specific genetic markers of trainability is therefore central to understanding this interaction.

A prime candidate gene in this context is the vitamin D receptor (*VDR*) gene [5], which encodes the receptor protein that mediates vitamin D’s biological actions. Functioning as a ligand-activated transcription factor, *VDR* is a pivotal regulator of skeletal muscle and bone physiology [6]. It orchestrates calcium-phosphate homeostasis, muscle protein synthesis, and bone metabolism through both genomic and rapid non-genomic signaling pathways [7]. The *VDR* gene harbors several common single nucleotide polymorphisms (SNPs), notably *FokI* (rs2228570), *BsmI* (rs1544410), *ApaI* (rs7975232), and *TaqI* (rs731236). These SNPs can modulate *VDR* function by altering protein activity, mRNA stability, or gene expression, thereby creating inter-individual differences in *VDR* signaling efficacy [8,9,10]. This functional variability provides a plausible mechanistic basis for the genetic contribution to muscle and bone-related traits [8].

While cross-sectional studies have reported associations between these *VDR* polymorphisms and baseline measures of muscle function and bone density [11,12], a critical evidence gap exists in longitudinal, intervention-based research [8]. The current literature is predominantly derived from associative studies in Caucasian populations, highlighting two specific shortcomings: first, a paucity of prospective studies examining whether *VDR* genotypes can predict the magnitude of adaptive gains to controlled resistance training; and second, a notable lack of such investigation in Chinese Han populations, where allele frequencies and gene-environment interactions may differ [13,14].

To address these gaps, this study employed a 12-week standardized resistance training intervention to investigate the association between key *VDR* polymorphisms and inter-individual differences in training adaptation among healthy Chinese Han adults. We hypothesized that distinct genotypes would be associated with significant differences in the training-induced response rates of muscle strength, muscle mass, and bone mineral content. The findings aim to provide novel insights into the genetic architecture of exercise adaptation in this population and contribute to the foundational knowledge for developing genetically informed, personalized exercise recommendations.

## 2. Materials and Methods

### 2.1. Study Participants and Ethical Approval

Participants: Undergraduate students from non-sports majors were recruited as participants. All were required to have had no systematic resistance training experience within the three months prior to this study. Individuals with risk conditions such as hypertension or heart disease were excluded. Initially, 216 participants were enrolled and completed baseline testing. During the intervention period, 25 participants withdrew or failed to complete the required training sessions, resulting in an attrition rate of 11.6%. The final sample consisted of 191 participants (95 males, 96 females). The progression of squat and bench press One-Repetition Maximum(1RM) loads throughout the intervention period is presented in Supplementary Figure S1. All participants were of Han Chinese ethnicity, aged 18–30 years, and were naturally grouped according to genotype. Genotyping was performed after the completion of all training interventions and phenotypic tests, and all research staff and analysts were blinded to the participants’ genotypes during the testing and intervention phases. During the formal intervention period, participants were instructed not to engage in any additional strength training outside of the experimental protocol. The testing and intervention periods of this study spanned autumn and winter (September to December; 12~26 °C), during which the participants were exposed to natural sunlight. All participants were advised to maintain their routine daily activities, and no special control over sunlight exposure duration or vitamin D supplementation was conducted. Diet, sleep, and other daily routines remained unchanged. Furthermore, participants were prohibited from consuming sports nutrition supplements (e.g., protein powder) or any special medications that could influence the experimental outcomes. The basic characteristics of the participants are presented in Table 1.

Ethical Approval and Informed Consent: See the Afterword.

### 2.2. Intervention Protocol

The intervention consisted of two exercises: the squat and the bench press. The training protocol was set at 10 repetitions × 5 sets per exercise, using a load corresponding to 70% of the individual’s one-repetition maximum (1RM). A rest interval of 2 min was allotted between sets. Training sessions were conducted twice weekly for a total duration of 12 weeks. To account for strength gains, 1RM tests were re-conducted every four weeks to adjust the training loads accordingly.

Prior to the formal intervention, one instructional session was held to ensure proper exercise technique. During formal training, participants were divided into small groups of 12 for supervision. To ensure adherence and allow for flexible scheduling, participants were divided into three training groups (training on Monday/Thursday, Tuesday/Friday, or Wednesday/Saturday). Each session was supervised by at least four certified trainers. A structured make-up policy was implemented: if a participant missed a scheduled session, they were required to attend a make-up session with another group within the same training week, maintaining a minimum 24 h recovery interval between sessions. Participants who failed to complete any session according to this protocol were excluded from the final analysis. Consequently, all included participants completed 100% (24 out of 24) of the prescribed training sessions. Under the premise of safety, participants were instructed to exert maximal effort in each set. If a participant was unable to complete all repetitions independently, a trainer provided minimal assistance. Three qualified trainers supervised and coached each group to ensure correct execution of every movement.

### 2.3. Assessment Measures

#### 2.3.1. Squat and Bench Press 1RM Strength Testing

The 1RM tests for the squat and bench press were performed on a Smith machine using a competition-grade barbell and standardized weight plates, with a precision of 0.5 kg. Tests were supervised and spotted by certified fitness professionals. Participants were required to be in good physical condition on the test day. The specific testing procedure followed established guidelines [15]. It is noteworthy that 1RM assessments were re-evaluated monthly.

#### 2.3.2. Lower Limb Isokinetic and Isometric Leg Press Strength

Isokinetic and isometric leg press strength were assessed using a German-manufactured ISOMED2000 (D&R Ferstl GmbH, Hemau, Germany) testing and training system. Prior to each test, participants performed a standardized warm-up, including 200 m of light jogging and two sets of 10 walking lunges, to increase hip and knee joint mobility and activate the lower limb musculature. After a brief period of free movement, participants entered the testing environment. The isokinetic test was administered first, followed by the isometric test after a 5 min rest interval.

For the isokinetic test, the pedal velocity was set at 100 cm/s. A large goniometer was used to define joint angles: 120° between the shank and thigh as the maximum extension position, and 90° between both the trunk and thigh and the shank and thigh as the maximum flexion position. Limit screws were placed on the device to prevent hyperextension or hyperflexion. Participants were given three practice trials before the formal test. After a 3 min rest, the formal test began with the pedal starting at the 120° knee extension position. Participants performed three consecutive maximal efforts, executing an eccentric contraction followed by a concentric contraction.

For the isometric test, the pedal was fixed at a knee angle of 120°. Participants exerted maximal force against the static pedal for 10 s. A 2 min rest was provided between successive trials. Test administrators provided standardized verbal encouragement during both pre- and post-intervention tests to maintain consistent motivation and minimize bias from varying arousal levels.

In the muscle strength and power tests, the highest value among three attempts was adopted for analysis, which was designed to reflect an individual’s optimal neuromuscular activation capacity rather than the average performance level.

#### 2.3.3. Countermovement Jump (CMJ) Height and Peak Power

A portable three-dimensional force platform (Kistler AG, Winterthur, Switzerland) was used to assess the countermovement jump (CMJ) height and peak power. Participants performed a maximal-effort CMJ starting from a standing position. Upon the tester’s command, they rapidly descended to a position where the thighs were approximately parallel to the ground and then immediately jumped vertically with maximal effort. Participants were instructed to maintain an upright torso during the entire movement, avoid bending the knees during the flight phase, and land with a controlled absorption. The test was performed three times. Jump height and peak power were derived from the force-time data using dedicated software, and the highest value was used for analysis.

#### 2.3.4. Body Composition via Dual-Energy X-Ray Absorptiometry (DEXA)

Body composition was assessed using a GE Lunar iDXA densitometer (GE Healthcare, Madison, WI, USA). Measurements were performed in standard scanning mode following the manufacturer’s guidelines. The machine was calibrated prior to testing. Participants were tested in a fasting state after removing outer clothing, metal belts, jewelry, and other high-density objects. They were positioned supine within the scanning area with palms resting naturally on the thighs, ensuring the entire body remained within the scan boundaries. The scan duration was approximately 7 min, depending on height.

#### 2.3.5. Muscle Thickness via Ultrasonography

Muscle thickness of the pectoralis major, rectus femoris, and vastus intermedius was measured bilaterally using a LOGIQ color Doppler ultrasound system (GE Healthcare, Madison, WI, USA). Each muscle site was measured three times per side, and the average value for each side was calculated; the overall mean of the left and right sides was then determined. The specific measurement protocol followed established guidelines [16]. All pre- and post-intervention measurements were performed by the same technician to ensure consistency.

### 2.4. Laboratory Methods

#### 2.4.1. Instruments and Reagents

Major Instruments: Forma −86 °C ultra-low temperature freezer (Thermo Electron, Waltham, MA, USA), laboratory refrigerator (Siemens, Munich, Germany), standard constant-temperature water bath, ES-315 vertical autoclave (TOMY-KOGYO, Tokorozawa, Saitama, Japan), TGuide M16 automated nucleic acid extraction system, vortex mixer, high-speed centrifuge, CGA genotyping chip (Illumina, San Diego, CA, USA).

Major Reagents: TGuide 1.2 mL large-volume blood genomic DNA extraction kit (TIANGEN, Beijing, China), isopropanol, ethanol, etc.

#### 2.4.2. DNA Extraction

Blood Sample Collection and Storage: To avoid the acute effects of resistance training, venous blood draws were scheduled prior to strength testing sessions. Participants refrained from vigorous exercise for one week and from moderate-to-high intensity exercise for three days prior to sampling. They maintained normal sleep patterns and fasted for 12 h. Blood (5 mL) was drawn from the antecubital vein into EDTA anticoagulant tubes. Samples were stored at 2–8 °C for up to two months or at −80 °C for long-term preservation. All blood draws were performed by a designated phlebotomist.

DNA Extraction Procedure: Genomic DNA was extracted from peripheral blood leukocytes using a TIANGEN DNA extraction kit (TIANGEN, Beijing, CHN) according to the manufacturer’s instructions. DNA concentration and purity were measured using a NanoDrop2000 spectrophotometer (Thermo Fisher Scientific, Waltham, MA, USA). A concentration ≥ 20 ng/µL was deemed acceptable. Purity was assessed by the OD260/OD280 ratio, with an acceptable range of 1.75 to 1.90. A ratio below 1.75 indicated protein or other contamination, while a ratio above 1.90 suggested significant nucleic acid degradation. Samples failing these criteria were re-extracted. Qualified DNA samples were stored at −20 °C for subsequent analysis.

#### 2.4.3. Genotyping

Genotyping for the target polymorphisms was performed using a gene chip scanning method based on nucleic acid hybridization technology [17]. The service was conducted by Guangzhou Jingke Biotechnology Co., Ltd. (Guangzhou, China) using an Illumina CGA chip. Briefly, high-density probe sequences are immobilized on a substrate in a specific order and hybridized with labeled sample DNA. The hybridization signal intensity, detected via laser-induced fluorescence, determines the allelic status at each locus. The chip fabrication involves technologies such as in situ probe synthesis, photolithography, and confocal laser scanning microscopy.

### 2.5. Statistical Analysis

Statistical analyses were performed using SPSS software (version 21.0). Continuous data are presented as mean ± standard deviation. For indicators involving both the left and right sides, the mean value was calculated; an explanation was provided if significant differences were observed in a single side. Allele and genotype frequencies were tested for deviation from Hardy–Weinberg equilibrium using the chi-square test to assess the sample’s population representativeness.

Participants were naturally grouped according to genotype for analysis. The primary outcome for assessing training response was the percentage change: [(post-intervention value−baseline value)/baseline value × 100%], used to minimize the influence of baseline differences. Differences between genotype groups in baseline values, post-intervention values, and percentage changes were analyzed using a one-way analysis of variance (ANOVA) followed by the Least Significant Difference (LSD) post hoc test for multi-group comparisons, or an independent samples *t*-test for two-group comparisons.

All continuous data were first tested for normality using the Shapiro–Wilk test. For data conforming to normality and homogeneity of variance, parametric tests (ANOVA, *t*-test) were applied. Non-normally distributed data were analyzed using non-parametric tests (Kruskal–Wallis test or Mann–Whitney U test). The significance level was set at *p* < 0.05, and a highly significant level at *p* < 0.01.

Multiple Testing Correction: To control for the inflation of Type I error due to multiple comparisons across the primary hypotheses, the False Discovery Rate (FDR) was applied using the Benjamini-Hochberg procedure. The FDR correction was independently applied to the sets of *p*-values generated from the post hoc pairwise comparisons within each of the following three analysis tiers: Overall genotype effect: Comparisons of post-intervention absolute values between genotype groups for each locus. Sex-stratified genotype effect: Comparisons of post-intervention absolute values between genotype groups within each sex. Percentage change (Δ%) analysis: Comparisons of Δ% values between genotype groups in the overall cohort and in sex-stratified analyses. Baseline comparisons and within-group (pre- vs. post-intervention) comparisons were not subject to FDR correction. All *p*-values reported in the results are uncorrected *p*-values. In line with a conservative approach, *p*-values between 0.04 and 0.05 are interpreted as indicating a statistical trend.

## 3. Results

### 3.1. Genotype Distribution of Candidate Loci

The distribution of genotypes and alleles for the *VDR* gene polymorphisms rs731236 (*TaqI*), rs7975232 (*ApaI*), and rs1544410 (*BsmI*) in the study population, along with the Hardy–Weinberg equilibrium (H–WE) test results, is presented in Table 2.

A comparison of allele frequencies between our cohort and the East Asian reference population in the gnomAD database revealed no significant differences (all *p* > 0.05, see Supplementary Table S1), confirming the genetic representativeness of our sample for the Han Chinese population.

### 3.2. Association of Candidate Loci with Resistance Training Responsiveness

Table 3 presents the association analysis between three *VDR* gene polymorphisms (rs731236, rs7975232, rs1544410) and selected training response indicators, including baseline values, post-intervention values, and between-group comparisons. Complete data are available in Supplementary Table S2.

**Table 3:** Dataset of *VDR* Gene Polymorphism and Resistance Training Response Outcomes in Total Participants.

Testing Metric	Genotype Group	Pre (Mean ± SD)	Post (Mean ± SD)	*p*-Value (Post Group Diff)
**rs731236 (******TaqI******)**				
**Muscle Strength**				
Back Squat 1RM (kg)	AA (*n* = 170)	76.33 ± 31.03	113.09 ± 38.06 ^▲▲^	>0.05
	AG (*n* = 21)	87.57 ± 29.04	125.33 ± 33.70 ^▲▲^	
Bench Press 1RM (kg)	AA (*n* = 170)	39.40 ± 20.04	54.72 ± 22.89 ^▲▲^	>0.05
	AG (*n* = 21)	48.10 ± 22.05	62.75 ± 22.87 ^▲▲^	
**Body Composition**				
Upper Limb Bone Mineral Content (g)	AA (*n* = 170)	168.60 ± 43.23	163.43 ± 44.65	0.014 *
	AG (*n* = 21)	181.23 ± 38.95	188.79 ± 46.24	
**rs7975232 (******ApaI******)**				
**Muscle Strength**				
Back Squat 1RM (kg)	AA (*n* = 10)	77.34 ± 36.75	115.90 ± 42.94 ^▲▲^	>0.05
	AC (*n* = 72)	79.94 ± 28.17	118.51 ± 35.90 ^▲▲^	
	CC (*n* = 109)	75.90 ± 32.22	111.47 ± 38.35 ^▲▲^	
Bench Press 1RM (kg)	AA (*n* = 10)	36.26 ± 21.95	51.90 ± 24.03 ^▲▲^	>0.05
	AC (*n* = 72)	42.35 ± 19.99	57.91 ± 22.30 ^▲▲^	
	CC (*n* = 109)	39.36 ± 20.47	54.40 ± 23.27 ^▲▲^	
**Body Composition**				
Upper Limb Bone Mineral Content (g)	AA (*n* = 10)	156.80 ± 44.53	160.05 ± 46.52	>0.05
	AC (*n* = 72)	168.38 ± 44.39	171.07 ± 48.65 ^▲^	
	CC (*n* = 109)	162.91 ± 4227	163.58 ± 43.18	
**rs1544410 (******BsmI******)**				
**Muscle Strength**				
Back Squat 1RM (kg)	CC (*n* = 169)	76.40 ± 31.01	113.16 ± 38.06 ^▲▲^	>0.05
	CT (*n* = 22)	86.55 ± 29.60	124.27 ± 34.14 ^▲▲^	
Bench Press 1RM (kg)	CC (*n* = 169)	39.49 ± 20.00	54.83 ± 22.81 ^▲▲^	>0.05
	CT (*n* = 22)	46.95 ± 22.60	61.95 ± 23.70 ^▲▲^	
**Body Composition**				
Upper Limb Bone Mineral Content (g)	CC (*n* = 169)	162.75 ± 43.31	163.58 ± 44.76	0.026 *
	CT (*n* = 22)	179.23 ± 39.15	186.50 ± 46.21	

Note: ^▲^ indicates a significant within-group difference pre- vs. post-intervention with *p* < 0.05; ^▲▲^ indicates *p* < 0.01. * indicates significant differences among different genotype groups post-intervention *p* < 0.05. Bold text denotes different loci and types of test indicators.

This study systematically analyzed the association between three *VDR* gene polymorphisms (rs731236, rs7975232, rs1544410) and resistance training responsiveness. Prior to the intervention, no statistically significant differences were observed among genotype groups across all measured indicators of muscle strength, power, body composition, or muscle thickness (all *p* > 0.05), confirming comparable baseline characteristics. Following the 12-week resistance training intervention, all genotype groups exhibited significant within-group improvements in the majority of indicators (most *p* < 0.01), validating the effectiveness of the training protocol (detailed data are provided in Supplementary Table S2).

Between-group comparisons revealed heterogeneity in the influence of genotypes across different loci on training adaptations:

rs731236 (*TaqI*): Carriers of the AG genotype demonstrated a significantly greater absolute gain in upper limb bone mineral content compared to AA genotype carriers (188.79 ± 46.24 g vs. 163.43 ± 44.65 g, *p* < 0.05). However, no statistically significant between-group differences were observed for key indicators of maximal upper- and lower-body strength (squat and bench press 1RM), lower limb isokinetic/isometric strength, or total body lean mass (all *p* > 0.05), although the AG group generally showed higher numerical values for most indicators.

rs1544410 (*BsmI*): Similarly, CT genotype carriers showed a significantly greater absolute gain in upper limb bone mineral content compared to CC genotype carriers (186.50 ± 46.21 g vs. 163.58 ± 44.76 g, *p* < 0.05), but their influence on maximal strength indicators did not reach statistical significance.

rs7975232 (*ApaI*): No statistically significant differences were found among the three genotypes (AA, AC, CC) for any of the tested indicators following the intervention (all *p* > 0.05), suggesting a limited impact of this polymorphism on the overall training response within the present cohort.

### 3.3. Stratified Association Analysis by Sex

To further investigate potential sex-specific effects of *VDR* polymorphisms on training response, stratified analyses were conducted for male and female participants separately. The results indicated heterogeneity in the association patterns between genotypes and training outcomes across sexes.

#### 3.3.1. Association of *VDR* Polymorphisms with Training Outcomes in Female Participants

In females, significant associations between genotypes and specific metrics were observed both at baseline and post-intervention, with the patterns differing between time points.

Baseline Differences: Prior to training, the rs1544410 polymorphism showed associations with baseline lower limb isokinetic strength in females (see Table 4). Specifically, females with the CT genotype had significantly higher baseline values for isokinetic flexion total work (813.00 ± 284.44 J vs. 636.86 ± 206.37 J, *p* = 0.028), average power (271.25 ± 94.69 W vs. 212.63 ± 68.77 W, *p* = 0.028) compared to the CC genotype, peak power (294.75 ± 104.15 W vs. 235.01 ± 77.56 W, *p* = 0.046) was greater in the CT group though this difference should be interpreted with caution given its borderline significance. suggesting a potential advantage in baseline lower limb flexion strength for CT carriers. No significant baseline differences were found for the rs731236 polymorphism.

Post-Intervention Response Differences: After 12 weeks, the association patterns shifted, primarily involving the rs731236 and rs1544410 loci (see Table 5).

**Table 5:** Association Analysis between *VDR* Gene Polymorphisms and Training Outcome-Related Indicators after Resistance Intervention in Females.

Check Point	Test Metrics	Genotype		t	*p*
rs731236		AA	AG		
	Isokinetic Flexion Average Work (J)	205.15 ± 69.53	188.14 ± 61.70	2.228	0.028 *
	Isokinetic Extension Average Work (J)	208.88 ± 65.31	198.29 ± 56.45	2.022	0.046 *
	Isometric Leg Press Peak Force (N)	2809.48 ± 965.95	2521.00 ± 828.40	2.071	0.041 *
	Vertical Jump Relative Peak Power (W/kg)	19.72 ± 3.52	16.91 ± 2.22	2.070	0.041 *
rs1544410		CC	CT		
	Vertical Jump Relative Peak Power (W/kg)	19.81 ± 3.48	16.35 ± 2.20	2.754	0.007 **

Note: * indicates a significant difference between groups with *p* < 0.05; ** indicates *p* < 0.01.

rs731236: Contrary to the baseline pattern, post-training, females with the AA genotype demonstrated superior performance compared to the AG genotype in isokinetic flexion average power (205.15 ± 69.53 J vs. 188.14 ± 61.70 J, *p* = 0.028), extension average power (208.88 ± 65.31 J vs. 198.29 ± 56.45 J, *p* = 0.046), and peak isometric leg press force (2809.48 ± 965.95 N vs. 2521.00 ± 828.40 N, *p* = 0.041). The AA genotype also showed a higher relative maximal power during the vertical jump test (19.72 ± 3.52 W/kg vs. 16.91 ± 2.22 W/kg, *p* = 0.041).

rs1544410: A reversal of the baseline advantage was also observed for this locus. Post-intervention, females with the CC genotype exhibited a significantly higher relative maximal power than those with the CT genotype (19.81 ± 3.48 W/kg vs. 16.35 ± 2.20 W/kg, *p* = 0.007). No significant differences were found for other indicators.

In summary, within the female cohort, the association between *VDR* polymorphisms and strength phenotypes appeared time-dependent. The CT genotype at rs1544410 may be linked to better baseline flexion strength, whereas after systematic training, the AA genotype at rs731236 and the CC genotype at rs1544410 were associated with more favorable training adaptations.

#### 3.3.2. Association of *VDR* Polymorphisms with Training Outcomes in Male Participants

In males, no significant between-genotype differences were observed at baseline for any locus. Post-intervention differences emerged for a few indicators, primarily related to muscle morphology for the rs7975232 and rs1544410 loci (see Table 6).

**Table 6:** Association Analysis between *VDR* Gene Polymorphisms and Training Outcome-Related Indicators after Resistance Intervention in Males.

Check Point	Test Metrics	Genotype		t	*p*
rs7975232		AA + AC ^a^	CC		
	Combined Thickness of Rectus Femoris and Vastus Intermedius Muscles (cm)	4.70 ± 0.93	5.09 ± 0.75	−2.209	0.040 *
	Isokinetic Extension Average Work (J)	296.17 ± 110.48	372.91 ± 147.82	−2.673	0.009 **
rs1544410		CC	CT		
	Combined Thickness of Left Rectus Femoris and Vastus Intermedius Muscles (cm)	4.98 ± 0.63	4.80 ± 0.55	2.253	0.027 *
	Combined Thickness of Rectus Femoris and Vastus Intermedius Muscles (cm)	4.96 ± 0.71	4.85 ± 0.52	0.527	0.599

Note: * indicates a significant difference between groups with *p* < 0.05; ** indicates *p* < 0.01; ^a^: the AA and AC genotypes were pooled into a single group for analysis to avoid statistical bias arising from the low number of AA genotype carriers.

rs7975232: Significant differences among genotypes were found for the thickness of the rectus femoris and vastus intermedius (CC: 5.09 ± 0.75 cm; AA + AC: 4.70 ± 0.93 cm; *p* = 0.040). Furthermore, for lower limb isokinetic extension average power, the CC genotype demonstrated higher values than the AA + AC genotype (372.91 ± 147.82 J vs. 296.17 ± 110.48 J, *p* = 0.009).

rs1544410: A significant association was found in males for the thickness of the left rectus femoris and vastus intermedius, with the CC genotype showing greater values than the CT genotype (4.98 ± 0.63 cm vs. 4.80 ± 0.55 cm, *p* = 0.027), but there was no significant difference in the mean values of the rectus femoris and vastus intermedius muscles between the left and right sides, suggesting a potential link to lower limb muscle hypertrophy in males.

In summary, sex-stratified analysis revealed association patterns that differed from the overall analysis. In females, the AA genotype at rs731236 was associated with superior isokinetic flexion average power (*p* < 0.05), whereas the AG genotype, which showed an advantage in the overall analysis, did not exhibit this association. Similarly, in males, the CC genotype at rs1544410 was linked to greater gains in isokinetic extension average power and quadriceps thickness (*p* < 0.05), contrasting with the overall analysis where the CT genotype appeared more advantageous. These results suggest that the influence of *VDR* polymorphisms on resistance training outcomes may be sex-specific.

### 3.4. Association Analysis Based on Percentage Change (Δ%)

To more accurately assess training-induced physiological changes while controlling for baseline variation, the percentage change (Δ%) for each indicator was calculated and analyzed.

#### 3.4.1. Overall Percentage Change Analysis

**Figure 1:**
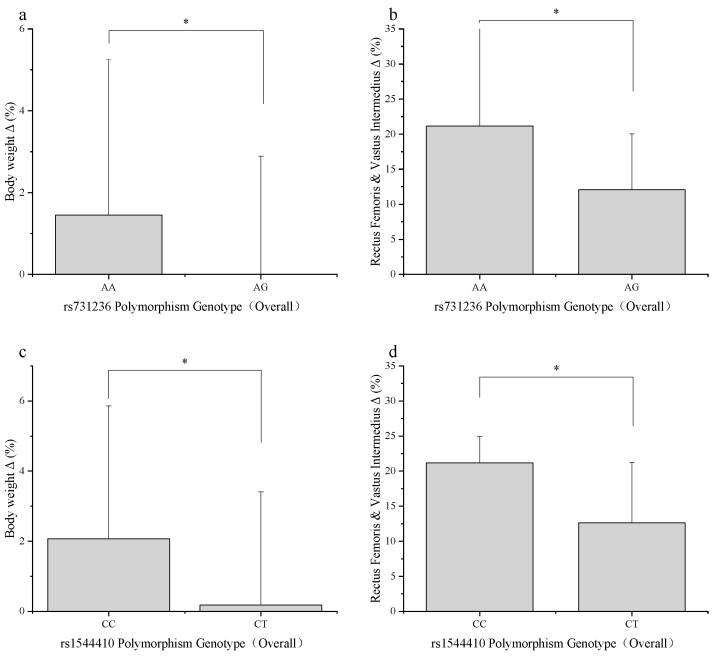
Association between *VDR* gene polymorphisms and overall training effect percentage change (Δ%). (**a**) Comparison of percentage change in body weight between different genotypes (AA vs. AG) at the rs731236 locus. (**b**) Comparison of percentage change in the combined thickness of the rectus femoris and vastus intermedius muscles between genotypes (AA vs. AG) at the rs731236 locus. The AG genotype showed significantly lower percentage gains in both indicators compared to the AA genotype (*p* < 0.05). (**c**) Comparison of percentage change in body weight between different genotypes (CC vs. CT) at the rs1544410 locus. (**d**) Comparison of percentage change in the combined thickness of the rectus femoris and vastus intermedius muscles between genotypes (CC vs. CT) at the rs1544410 locus. The CT genotype showed significantly lower percentage gains in both indicators compared to the CC genotype (*p* < 0.05). Data are presented as mean ± standard deviation. * indicates a statistically significant between-group difference (*p* < 0.05).

In the entire sample, only the rs731236 and rs1544410 loci showed significant associations with the percentage change in a few indicators (see Figure 1).

rs731236: The AG genotype showed a significantly lower percentage change in body weight compared to the AA genotype (−0.09 ± 2.98% vs. 1.45 ± 3.81%, *p* = 0.027). The AG genotype also exhibited a significantly lower percentage increase in the thickness of the rectus femoris and vastus intermedius (21.15 ± 17.14% vs. 12.10 ± 7.97%, *p* = 0.018).

rs1544410: The CT genotype showed significantly lower percentage changes compared to the CC genotype in both body weight (0.18 ± 3.23% vs. 2.07 ± 3.79%, *p* = 0.027) and the thickness of the rectus femoris and vastus intermedius (21.16 ± 17.18% vs. 12.63 ± 8.61%, *p* = 0.023).

#### 3.4.2. Sex-Stratified Percentage Change Analysis

**Figure 2:**
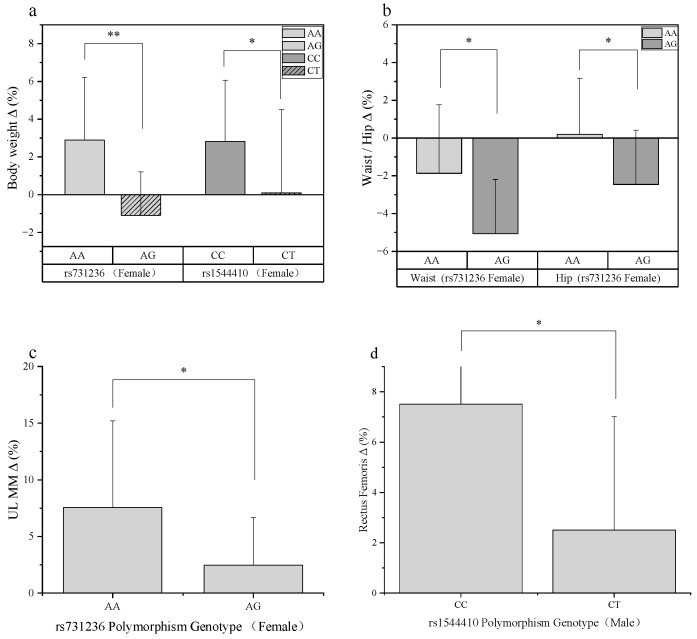
Association between *VDR* gene polymorphisms and training effect percentage change (Δ%) stratified by sex. (**a**) In females, the percentage change in body weight was significantly lower in carriers of the AG genotype at the rs731236 locus compared to AA genotype carriers (*p* < 0.01); the percentage change in body weight was significantly lower in carriers of the CT genotype at the rs1544410 locus compared to CC genotype carriers (*p* < 0.05). (**b**) In females, the absolute values of percentage change in waist circumference (left) and hip circumference (right) were significantly greater in carriers of the AG genotype at the rs731236 locus compared to AA genotype carriers (*p* < 0.05). (**c**) In females, the change rate of upper limb muscle mass (UL MM) was significantly lower in carriers of the AG genotype at the rs731236 locus compared to AA genotype carriers (*p* < 0.05). (**d**) In males, the percentage increase in the combined thickness of the rectus femoris muscles was significantly lower in carriers of the CT genotype at the rs1544410 locus compared to CC genotype carriers (*p* < 0.05). Data are presented as mean ± standard deviation. * indicates a statistically significant between-group difference (*p* < 0.05), and ** indicates *p* < 0.01.

In females, the rs731236 and rs1544410 loci were significantly associated with changes in body composition metrics (see Figure 2a–c).

rs731236: Females with the AG genotype showed significantly lower percentage changes than those with the AA genotype in body weight (−1.09 ± 2.30% vs. 2.89 ± 3.32%, *p* = 0.003), waist circumference (−5.07 ± 2.88% vs. −1.87 ± 3.63%, *p* = 0.025), hip circumference (−2.46 ± 2.88% vs. 0.20 ± 2.97%, *p* = 0.024), and upper limb muscle mass (2.47 ± 4.22% vs. 7.55 ± 7.67%, *p* = 0.020).

rs1544410: Females with the CT genotype had a significantly lower percentage change in body weight compared to those with the CC genotype (0.11 ± 4.40% vs. 2.82 ± 3.24%, *p* = 0.031).

Notably, for the rs731236 locus, the mean percentage changes for the AG genotype were negative for body weight, waist, and hip circumference, while for the AA genotype, only the mean waist circumference change was negative. This pattern was not observed for other genotypes in either sex.

In Males: Only the rs1544410 locus showed a significant association, specifically with the percentage change in the thickness of the rectus femoris (see Figure 2d). The increase was significantly lower in CT genotype males compared to CC genotype males (7.51 ± 8.40% vs. 2.51 ± 7.04%, *p* = 0.025).

## 4. Discussion

Vitamin D receptor (*VDR*) gene polymorphisms have long been implicated in phenotypes related to muscle function, bone metabolism, and body composition [8]. In recent years, with advancements in exercise genomics, the *VDR* gene has gained attention as a key candidate influencing individual training responsiveness [18,19,20]. While existing studies have explored associations between *VDR* polymorphisms and baseline muscle strength and bone mineral density [9], their dynamic roles during resistance training interventions and sex-specific responses remain unclear. This study, through a 12-week standardized resistance training intervention, systematically analyzed the associations of three common *VDR* polymorphisms (rs731236, rs7975232, rs1544410) with training responses (muscle strength, morphology, bone mineral content) in Chinese Han adults. Furthermore, it explored sex differences, providing a genetic basis for personalized exercise prescription.

### 4.1. Analysis of Genotype Distribution for Candidate Loci

In this study, the genotype distributions for rs731236 (*TaqI*), rs7975232 (*ApaI*), and rs1544410 (*BsmI*) all conformed to Hardy–Weinberg equilibrium (H–WE) (*p* > 0.05), indicating that the sample is population-representative and suitable for genetic association analysis. In contrast, the rs2228570 (*FokI*) locus significantly deviated from H–WE (*p* < 0.05). This suggests potential selection bias in the participant cohort or a possible genotype-specific survival effect at this locus, consistent with findings in some other studies [21,22]. This deviation may arise from multiple factors, including random fluctuations in the specific sample of the present study, potential genotyping errors, or selection pressure acting on the locus itself within the population. We opted to exclude it in accordance with methodological conventions to ensure the validity of the association analysis. It suggests the need for larger samples or multi-ethnic validation in future research.

### 4.2. Association Analysis of rs731236 (TaqI) Polymorphism with Resistance Training Responsiveness

Consistent with the conclusions of a systematic review in this field [8], our findings indicate that the association of the rs731236 polymorphism with muscle function is relatively limited, manifesting more prominently in bone metabolism and localized muscle morphological adaptation. rs731236 is located in exon 9 of the *VDR* gene and is a synonymous mutation that may regulate *VDR* function by affecting mRNA stability or translation efficiency. This study showed that in the overall sample, the rs731236 polymorphism was associated with training responses in specific muscle morphology indicators: AG genotype carriers showed significantly greater gains in upper limb bone mineral content and Combined Thickness of Rectus Femoris and Vastus Intermedius Muscles compared to AA genotype carriers (*p* < 0.05). However, in the percentage change analysis, which is more explanatory as it controls for baseline values, the AG genotype showed a significantly lower increase in the thickness of the rectus femoris and vastus intermedius compared to the AA genotype. In the female cohort, post-training, the AA genotype demonstrated superior performance in isokinetic strength and vertical jump power. The percentage change analysis in females further indicated that the AA genotype had a significantly greater increase in upper limb muscle mass (Ul MM), suggesting that females with the AA genotype are more responsive to resistance training in terms of muscle morphology. Conversely, the AG genotype showed weaker gains or even decreases in body composition indicators like body weight, waist circumference, and hip circumference. This suggests that resistance training may be more beneficial for fat loss and weight reduction in females with the AG genotype. These findings imply that rs731236 may influence the sensitivity of different tissues (bone vs. muscle) to training adaptation by modulating *VDR* expression efficiency, with effects being both sex- and phenotype-specific. Consistent with the systematic review by Krasniqi et al. [8], our study found the association of rs731236 with muscle function to be relatively limited, manifesting more in bone metabolism and localized muscle morphological adaptation.

#### 4.2.1. *VDR* Polymorphisms and Heterogeneous Responses in Muscle Performance

Our sex-stratified analysis revealed that in females, the AA genotype of rs731236 was associated with superior gains in isokinetic flexion/extension average power and isometric leg press force following training (see Table 5). Regarding muscle and strength, most existing studies report no differences in grip strength or isokinetic tests of knee extension and flexion among genotypes. Our results align with this for baseline values, showing no significant differences in any indicator for either sex [23,24]. However, post-intervention in females, the AA genotype was significantly superior to the AG genotype in isokinetic flexion/extension average power, isometric leg press force (at 120°), and relative maximal jump power. This may indicate that systematic resistance training potentiates more rapid strength development in AA genotype or A allele carriers, though caution is warranted in interpreting these findings given that some of the associated *p*-values were close to the significance threshold (e.g., *p* ≈ 0.04–0.05).

Research by Makanae Y et al. [25] using a rat model showed a significant immediate increase in *VDR* protein expression in the gastrocnemius muscle following resistance training, along with a rise in cytochrome P450 27B1, an enzyme involved in vitamin D metabolism. These changes were not observed in an endurance training group. Resistance training also increased phosphorylated ERK1/2 and Mnk1, which may be involved in regulating *VDR* expression. This study first demonstrated that resistance training is a more potent stimulus for elevating intramuscular *VDR* expression compared to endurance training. Another human study found a positive correlation between *VDR* mRNA expression levels and the degree of muscle hypertrophy after 20 weeks of resistance training [26]. These prior studies sufficiently demonstrate that resistance exercise can induce increased *VDR* expression, promoting skeletal muscle hypertrophy. However, the relationship between *VDR* gene polymorphisms and the expression of *VDR* protein and related molecules requires further investigation.

#### 4.2.2. *VDR* Polymorphisms and Divergent Adaptations in Body Composition

We observed that female carriers of the AG genotype (rs731236) exhibited significant reductions in body weight, waist, and hip circumference after training, in contrast to the AA genotype carriers who showed increases (Figure 2). This finding points to a potential role of the *VDR* polymorphism in modulating body composition adaptations to resistance exercise. Several studies suggest *VDR* gene polymorphisms have a potential role in obesity, being implicated in its pathogenesis, though the results are inconsistent [27]. In young males [28], the *TaqI* “tt” genotype was associated with higher BMI and waist circumference compared to other *TaqI* genotypes. In females [29], the “bAT” haplotype for *BsmI*, *ApaI*, and *TaqI* was negatively associated with central obesity. The *TaqI* “T” allele has been linked to increased obesity risk, and the “TT” genotype with obesity risk and weight gain [30]. Conversely, other studies found no significant difference in waist circumference or BMI between males and females due to the *TaqI* polymorphism [31,32]. Our results indicate that in female participants, the AG genotype (equivalent to the *TaqI* Tt genotype), due to the presence of the G allele, exhibited reductions in body weight, waist circumference, and hip circumference after 12 weeks of resistance training. This suggests that G allele carriers may be more resistant to obesity risk and weight gain. Since no significant differences existed between genotype groups before the intervention, this implies that certain gene expression effects may only manifest under the influence of specific behavioral interventions. Resistance training may confer weight loss benefits for G allele carriers, though this finding requires further validation with larger cohorts, inclusion of aerobic training interventions, and control of other confounding factors.

*VDR* gene expression occurs in adipose tissue, with studies finding reduced *VDR* expression in the adipose tissue of obese individuals [33]. *VDR* expression in adipose tissue confirms the key role of vitamin D in adipose tissue function [34]. *VDR* gene polymorphisms may influence vitamin D’s action in adipose tissue by affecting *VDR* structure and expression. Vitamin D can influence obesity development through multiple factors, including its roles in energy intake, cholesterol metabolism, regulation of lipase gene expression, and adipogenesis [35]. Resistance training, in turn, alters energy expenditure and affects adipose tissue metabolism [36]. As noted, the inconsistency across studies may be due to key factors such as diverse ethnicity, age, sex, and exercise interventions.

#### 4.2.3. Association Between *VDR* Polymorphisms and Bone Mineral Content Responses

Regarding bone mineral content, Although the present study measured bone mineral content (BMC), a mass measure, the widely reported associations between *VDR* polymorphisms and bone mineral density (BMD), an area-corrected density measure, are highly relevant [20,37]. Given that BMC and BMD are strongly correlated and respond similarly to mechanical loading [38], our findings of genotype-associated differences in BMC adaptation likely reflect analogous effects on bone density and quality. In the overall cohort, carriers of the AG genotype (rs731236) displayed significantly greater post-intervention right upper limb bone mineral content compared to AA carriers (Table 3). This aligns with the broader context of bone metabolism, where both mechanical loading and genetic factors, including *VDR* polymorphisms, are known to be key regulators [39]. A study in the Chinese Zhuang population found the GG genotype of rs2228570 associated with reduced bone mass risk and identified complete linkage disequilibrium between rs7975232 and rs731236 [40]. Such linked variants may synergistically affect bone metabolism. Regression analysis revealed that the *VDR* *FokI* GG genotype predicted lower bone mineral density of bone mineral density, whereas exercise intervention could ameliorate this adverse effect [41]. A meta-analysis concluded that the t allele at the *TaqI* locus was significantly correlated with an elevated risk of decreased bone mineral density (OR = 1.51, 95% CI: 1.32–1.72), which further confirms that *TaqI* polymorphism acts as a genetic factor regulating bone mineral deposition, with the t allele serving as a reliable genetic marker for predicting the risk of bone mineral density reduction [42]. Our results showed that post-intervention for rs731236, the AG genotype had significantly higher right upper limb bone mineral content than the AA genotype. However, this finding was not replicated in sex-stratified or percentage change analyses and therefore requires further verification.

Another intriguing finding is the inconsistency of the “advantageous genotype” for rs731236 between overall and sex-stratified analyses, strongly suggesting a genotype-sex interaction. Research confirms that *VDR* polymorphisms exhibit differences in baseline muscle strength and strength training response between sexes. The likely mechanism involves complex crosstalk between sex hormones (e.g., estrogen and testosterone) and the vitamin D signaling pathway, potentially modulating *VDR* expression or function, leading to different phenotypic outcomes for the same genetic variant in different physiological contexts [43]. Research by Dong-Ho Park et al. [44] showed that in females, subjects carrying the Ff heterozygous and ff homozygous genotypes had 5.2–6.7% higher grip strength than FF homozygotes, a statistically significant difference. In males, while no significant statistical difference in grip and back strength was found across *FokI* genotypes, strength indicators were still slightly higher in Ff and ff carriers than in FF homozygotes. A Swedish study focused on women found polymorphisms in the *VDR* gene polyadenylate repeat sequence and the *BsmI* locus were associated with muscle strength and fat mass [45]. Women carrying shorter poly-A repeats, the ss genotype, and the absence of the *BsmI* restriction site (bb) had higher hamstring strength, body weight, and fat mass than those with long repeats and the presence of the site. As this study focused on premenopausal women, it was speculated that sex hormones like estrogen might interact synergistically with these *VDR* genotypes to influence muscle strength and body composition, also hinting that the effect of this genotype might be more prominent in women, creating a potential sex difference compared to men. In men, sex hormones like testosterone participate in bone metabolism regulation. In one study, two male genotypes showed different changes in bone metabolism indicators post-training. The Ff/ff genotype group showed increased 1,25-dihydroxyvitamin D_3_ levels after training, while the FF group did not. However, the FF group exhibited more pronounced and longer-lasting inhibition of bone resorption. Testosterone may synergize with the *VDR* gene, affecting the metabolism of the active form of vitamin D, leading to differential bone metabolic responses to resistance training in men with different genotypes, reflecting the joint regulation of post-training bone metabolism by sex hormones and *VDR* genotype [46]. Our main finding is that for the rs731236 polymorphism, the AA genotype is associated with greater sensitivity to resistance training in terms of muscle strength and morphology in women.

Furthermore, inherent differences in baseline muscle mass, body composition, and bone density between men and women may lead to different starting points and potentials for training adaptation, thereby altering the direction and magnitude of genetic effects. However, the use of percentage change in this study controlled for baseline influences, making the analysis of resistance training sensitivity based on sex and percentage change more meaningful.

### 4.3. Association Analysis of rs7975232 (ApaI) Polymorphism with Resistance Training Responsiveness

rs7975232 (*ApaI*) is located in an intronic region, often in linkage disequilibrium with the *BsmI* locus, and may be involved in gene expression regulation [8]. The results of this study showed no significant between-group differences across genotypes (AA, AC, CC) for any muscle strength, power, or overall lean mass indicators before or after training, suggesting a weak influence on overall training adaptation. However, in males, the CC genotype was associated with significantly greater thickness of the rectus femoris and vastus intermedius compared to other genotypes, hinting at a potential role in sex-specific muscle morphological adaptation. No differences were found in the percentage change analysis for any indicators. Therefore, the association of the rs7975232 polymorphism with resistance training response remains to be determined. This aligns with inconsistent conclusions from previous research on the effect of *ApaI* polymorphism on bone density and muscle function, suggesting its effect may be modulated by factors like training type, ethnicity, and sex [14,47].

### 4.4. Association Analysis of rs1544410 (BsmI) Polymorphism with Resistance Training Responsiveness

rs1544410 (*BsmI*) is located in intron 8 of the *VDR* gene and may regulate *VDR* expression by affecting mRNA splicing or stability [48]. This study found that CT genotype carriers showed significantly greater post-training gains in upper limb bone mineral content compared to CC genotype carriers, suggesting this genotype may be more favorable for bone mineral accrual and morphological adaptation of specific muscle groups. Sex-stratified analysis further revealed that in females, the CT genotype was associated with higher baseline flexion strength, but post-training the advantage shifted to the CC genotype for power indicators. In males, particularly in the percentage change analysis, the CC genotype showed better performance in lower limb muscle thickness and isokinetic extension work. This “genotype-training intervention-sex” interaction suggests that the *BsmI* polymorphism may regulate muscle and bone adaptation mechanisms under different physiological states by influencing post-transcriptional regulation of *VDR*. T allele carriers may have slightly superior baseline muscle mass, strength, and bone mineral content compared to CC homozygotes, but after systematic resistance intervention, the CC genotype demonstrated better adaptive changes (though not for all indicators).

Our findings resonate with the systematic review by Krasniqi et al. [8], which emphasized that associations between *VDR* polymorphisms and muscle mass/function vary considerably across ethnicities and are moderated by vitamin D status. This underscores the importance of interpreting genetic associations within specific population and physiological contexts. More specifically, a study on Slovak postmenopausal women showed the *BsmI* polymorphism was associated with bone density and 25-hydroxyvitamin D levels, although its direct link to muscle adaptation was not clarified [19]. This suggests the *VDR* gene may exert influence by regulating common pathways in bone-muscle metabolism.

Mechanistically, rs1544410 is located in an intronic region of the *VDR* gene and may regulate *VDR* protein expression levels by affecting mRNA splicing efficiency or stability. Research by Bass et al. [26] provides a key clue, demonstrating that *VDR* overexpression is sufficient to activate the mTORC1 signaling pathway and induce skeletal muscle hypertrophy. Therefore, we hypothesize that genotypes associated with optimal *VDR* expression or activity (such as the CC type in this study) may more efficiently transduce vitamin D/anabolic signals after resistance training, promoting stronger protein synthesis and muscle growth. Furthermore, vitamin D and its receptor have been shown to regulate calcium homeostasis and satellite cell function within muscle, both key processes affecting muscle repair and growth [39,49]. Future research needs to directly measure *VDR* expression levels or the activity of downstream signaling pathways in participants before and after training to validate this hypothesis.

### 4.5. Comprehensive Mechanistic Discussion

The vitamin D receptor (*VDR*) serves as the central mediator of the vitamin D signaling pathway, regulating skeletal muscle protein synthesis, cell differentiation, and bone metabolism through both genomic and non-genomic mechanisms, thereby establishing the theoretical foundation for its influence on training adaptations [50]. The polymorphisms investigated in this study—rs1544410 (*BsmI*), rs731236 (*TaqI*), and rs7975232 (*ApaI*)—are all located within the 3’ region of the *VDR* gene and exhibit high linkage disequilibrium with polymorphisms in the 3’ untranslated region (3’ UTR) [10]. The differential associations we observed across loci, sexes, and phenotypes may originate from the distinct regulatory effects these polymorphisms exert on *VDR* expression, stability, or function.

Specifically, the rs731236 (*TaqI*) and rs1544410 (*BsmI*) polymorphisms may modulate *VDR* protein abundance by influencing mRNA secondary structure, splicing efficiency, or stability [8,51]. This provides a mechanistic framework for interpreting the phenotype-specific associations found in our study. In females, carriers of the rs731236-AA genotype demonstrated superior gains in muscle strength and power following resistance training. This advantage may be attributable to more efficient *VDR* protein translation associated with this genotype, leading to more effective activation of anabolic pathways such as mTOR and consequently enhancing post-training neuromuscular adaptation [51]. Conversely, the rs1544410 polymorphism showed a stronger association with adaptations in bone mineral content and specific muscle morphology (e.g., Rectus Femoris and Vastus Intermedius Muscles), suggesting its influence may be more oriented toward downstream pathways involved in tissue structure remodeling. Direct evidence supporting this notion comes from the work of Bass et al., who demonstrated that *VDR* overexpression in skeletal muscle is sufficient to activate the mTORC1 pathway and induce muscle hypertrophy [26]. This finding points to a potential common terminal pathway underlying the genotype-dependent muscular adaptations observed in our study. This mechanism is further corroborated by reverse evidence from Girgis et al., who reported that *VDR* knockout or vitamin D deficiency in a mouse model directly resulted in reduced grip strength, altered muscle fiber composition, and elevated myostatin levels [49]. Collectively, evidence from both directions solidifies the central role of *VDR* in regulating muscle mass and function.

In contrast, the intronic rs7975232 (*ApaI*) polymorphism did not exhibit widespread significant associations in our study, a finding consistent with the conclusions of most similar reports. Its functional impact may depend more on altering chromatin conformation or acting as an enhancer/silencer element to regulate *VDR* or neighboring gene expression in an allele-specific manner. Such effects might only become fully apparent in specific cell types or under particular environmental stimuli [52].

Another critical aspect revealed by our study is the significant genotype-by-sex interaction. A complex crosstalk exists between sex hormones (e.g., estrogen and testosterone) and the vitamin D signaling pathway [43,53]. For instance, estrogen can potentiate *VDR* transcriptional activity, while testosterone may influence the metabolism of active vitamin D [46,54,55]. Consequently, an identical *VDR* genotype may yield strikingly divergent molecular effects and phenotypic outcomes under the distinct hormonal milieus of males and females. This interaction aptly explains why the rs731236 AA genotype manifested as a “strength-advantageous” type in females, whereas the AG genotype was associated with greater bone mineral content gains in the overall analysis. This interplay underscores the necessity of stratifying analyses by sex as a key biological variable in exercise genetics research.

In summary, *VDR* gene polymorphisms may constitute a genetic network influencing inter-individual adaptability to resistance training. Different loci affect *VDR* functional dosage through diverse molecular mechanisms (transcriptional, post-transcriptional, and epigenetic regulation). These genetic effects are subsequently modulated by sex-specific hormonal environments, ultimately manifesting as heterogeneity in training responses across phenotypes such as muscle strength, body composition, and bone metabolism [43,56]. Future research should integrate tissue biopsies (e.g., from muscle and adipose tissue) obtained before and after exercise interventions. Direct assessment of dynamic changes in *VDR* expression and its downstream signaling pathways, coupled with systematic monitoring of circulating sex hormones and vitamin D levels, is warranted to empirically validate the comprehensive mechanistic framework proposed herein.

### 4.6. Research Implications and Future Directions

This study systematically explored the association between *VDR* gene polymorphisms and resistance training responses in a Chinese Han population. For the first time, it provided evidence for the longitudinal association between *VDR* genotypes and resistance training adaptability in this population, and revealed sex-specific adaptation patterns, providing preliminary evidence for “genotype-guided” personalized exercise recommendations. It further lays a preliminary conceptual framework and identifies candidate biomarkers for future exploration of “genotype-guided personalized training” while emphasizing that the current findings are preliminary and need to be verified in larger sample sizes and more diverse populations.

This study has limitations, such as a relatively limited sample size, inclusion of only young healthy individuals, and lack of concurrent vitamin D level monitoring. Due to the low minor allele frequency (MAF) of the key loci, the sample sizes of some genotype groups were relatively small, especially in the sex-stratified analyses. Therefore, the relevant findings should be regarded as exploratory, and their robustness requires verification in studies with larger sample sizes. Moreover, results with borderline statistical significance (*p* ≈ 0.04–0.05) should be interpreted conservatively and require further validation in independent and larger cohorts. Future research could combine vitamin D level monitoring, muscle biopsy and transcriptomic analysis, and exploration of genotype-sex interactions to further elucidate the molecular mechanisms by which *VDR* polymorphisms influence training adaptation. Furthermore, expanding sample size, extending intervention duration, and including multi-ethnic comparisons will help enhance the generalizability and application value of the conclusions.

## 5. Conclusions

This study systematically investigated the association between three *VDR* gene polymorphisms (rs731236, rs7975232, rs1544410) and resistance training benefit responses in Chinese Han adults through a 12-week standardized resistance training intervention. The main conclusions are as follows:

rs731236 (*TaqI*) locus: After a standardized resistance training intervention, individuals carrying the AG genotype in the overall cohort showed more significant gains in upper limb bone mineral accumulation. In addition, there was a significant gender dimorphism in training responses: among females, those with the AA genotype were more sensitive to improved strength and muscle mass from training, while those with the AG genotype showed greater fitness for training in terms of body composition optimization.

rs7975232 (*ApaI*) locus: It revealed no significant impact on overall training response, with only a limited association observed with lower limb muscle thickness in males.

rs1544410 (*BsmI*) locus: Following the resistance training intervention, individuals with the CC genotype were more responsive to the training stimulus, demonstrating greater adaptive benefits in both muscle power and (in males) lower limb muscle growth compared to other genotypes.

## Figures and Tables

**Table 1:** Basic characteristics of the participants.

Group	Number (*n*)	Height (cm)	Weight (kg)	Age (Years)
Total	191	171.45 ± 8.68	63.81 ± 13.16	20.84 ± 1.94
Male	95	178.10 ± 5.71	71.00 ± 12.44	20.42 ± 1.05
Female	96	165.01 ± 5.69	56.50 ± 9.21	21.24 ± 2.46

**Table 2:** Distribution of *VDR* Gene rs731236, rs7975232, rs1544410 Polymorphism and Hardy–Weinberg Equilibrium Test.

Locus	Sample Size (*n*)	Genotype (Distribution Frequency)			Allele (Distribution Frequency)		H–WE Test (χ^2^, *p* Value)	
rs731236 (*TaqI*)		AA	AG	GG	A	G	**χ^2^**	*p*
	Overall (191)	170 (0.89)	21 (0.11)	0 (0)	361 (0.95)	21 (0.05)	0.646	0.421
	Male (95)	81 (0.85)	14 (0.15)	0 (0)	176 (0.93)	14 (0.07)		
	Female (96)	89 (0.93)	7 (0.07)	0 (0)	185 (0.96)	7 (0.04)		
rs7975232 (*ApaI*)		AA	AC	CC	A	C	**χ^2^**	*p*
	Overall (191)	10 (0.05)	72 (0.38)	109 (0.57)	92 (0.24)	290 (0.76)	0.182	0.669
	Male (95)	4 (0.04)	43 (0.45)	48 (0.51)	51 (0.27)	139 (0.73)		
	Female (96)	6 (0.06)	29 (0.30)	61 (0.64)	41 (0.21)	151 (0.79)		
rs1544410 (*BsmI*)		CC	CT	TT	C	T	**χ^2^**	*p*
	Overall (191)	169 (0.88)	22 (0.12)	0 (0)	360 (0.94)	22 (0.06)	0.713	0.398
	Male (95)	81 (0.85)	14 (0.15)	0 (0)	176 (0.93)	14 (0.07)		
	Female (96)	88 (0.92)	8 (0.08)	0 (0)	184 (0.96)	8 (0.04)		

Chi-square tests on genotype distribution frequencies revealed that all three loci (rs731236: χ^2^ = 0.646, *p* = 0.421; rs7975232: χ^2^ = 0.182, *p* = 0.669; rs1544410: χ^2^ = 0.713, *p* = 0.398) conformed to H–WE (*p* > 0.05), indicating that the participant sample is representative of the population. In contrast, the genotype distribution for rs2228570 (*FokI*) (AA = 28, AG = 113, GG = 50) significantly deviated from H–WE (χ^2^ = 7.576, *p* = 0.006). Consequently, this locus was excluded from subsequent analyses.

**Table 4:** Association Analysis between *VDR* Gene Polymorphisms and Isokinetic Strength-Related Indicators at Baseline in Females.

Check Point	Test Metrics (J)	Genotype		t	*p*
rs1544410		CC	CT		
	Isokinetic Flexion Total Work	636.86 ± 206.37	813.00 ± 284.44	−2.238	0.028 *
	Isokinetic Flexion Average Work	212.63 ± 68.77	271.25 ± 94.69	−2.236	0.028 *
	Isokinetic Flexion Peak Work	235.01 ± 77.56	294.75 ± 104.15	−2.024	0.046 *

Note: * indicates a significant difference between groups with *p* < 0.05.

## Data Availability

The individual-level data generated and analyzed during this study are available from the corresponding author upon reasonable request. Due to privacy concerns and institutional ethical requirements, these data are not publicly available. All participants provided written informed consent, and data access is restricted to protect identifiable personal and genetic information. Access to all variables stratified by sex and genotype is temporarily restricted, as these data are undergoing further research and analysis.

## Supplementary Materials

The following supporting information can be downloaded at: https://www.mdpi.com/article/10.3390/genes17020137/s1, Supplementary Figure S1: Progression of training loads; Supplementary Table S1: Comparison of allele frequencies; Table S2: Dataset of *VDR* Gene Polymorphism (rs731236, rs7975232, rs1544410) and Resistance Training Response Outcomes in Total Participants.

## Abbreviations

The following abbreviations are used in this manuscript:

*VDR*	Vitamin D Receptor
SNP	Single Nucleotide Polymorphism
1RM	One-Repetition Maximum
CMJ	Countermovement Jump
DEXA	Dual-Energy X-ray Absorptiometry
FDR	False Discovery Rate
H–WE	Hardy–Weinberg Equilibrium
Δ%	Percentage Change
Ul MM	Upper Limb Muscle Mass
BMC	Bone Mineral Content
BMD	Bone Mineral Density
MAF	Minor Allele Frequency
