# Supplementary material for: VDR Gene Polymorphisms and Inter-Individual Variability in Response to Resistance Training
Source: Genes (Basel). 2026 Jan 27;17(2):137. doi: 10.3390/genes17020137 (PMC12940962; doi:10.3390/genes17020137)
Supplement: Supplementary file 1 [file genes-17-00137-s001.zip › genes-4115096-supplementary.pdf]

Supplementary Materials:

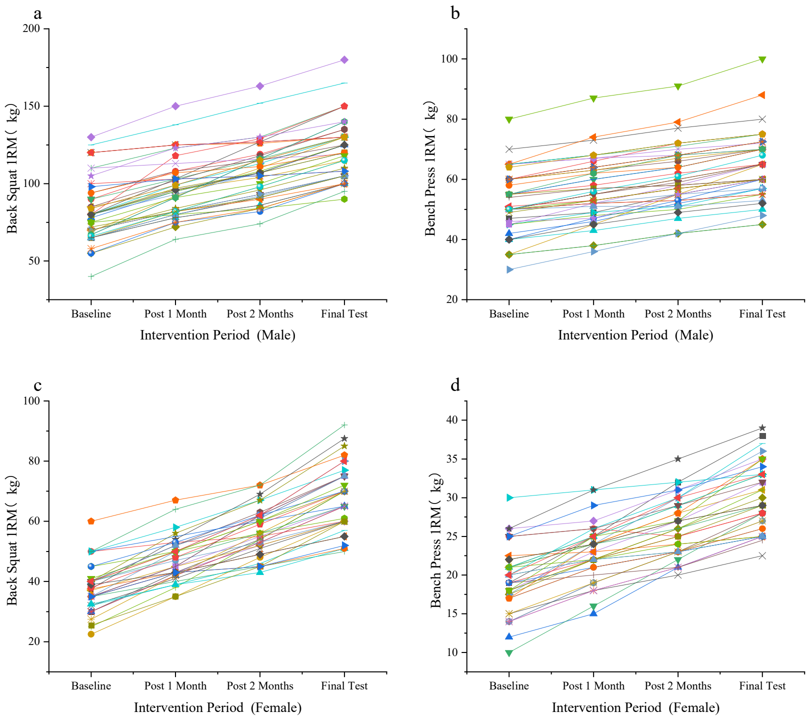

Figure S1. Progression of training loads throughout the 12-week resistance training intervention. The loads for the squat and bench press exercises are presented as absolute one-repetition maximum (1RM) values (kg), adjusted every 4 weeks based on re-assessed 1RM tests, with the first test representing the baseline value and the final test at the end of the 12-week intervention. **(a)** Change trend in squat 1RM for males; **(b)** Change trend in bench press 1RM for males; **(c)** Change trend in squat 1RM for females; **(d)** Change trend in bench press 1RM for females.

Table S1. Comparison of allele frequencies between the study cohort and the East Asian (EAS) reference population from gnomAD.

Check Point	Allele	Frequency in This Study (*N*=191)	Frequency in gnomAD EAS	*P* value
rs731236 (*TaqI*)	A	0.945	0.947	0.892
	G	0.055	0.053	
rs7975232(*ApaI*)	C	0.759	0.696	0.064
	A	0.241	0.304	
rs1544410(*BsmI*)	C	0.942	0.936	0.745
	T	0.058	0.064	
rs2228570 (*FokI*)	A	0.442	0.411	0.382
	G	0.557	0.589	

Note: Data Source: gnomAD (https://gnomad.broadinstitute.org/).

Table S2. Dataset of *VDR* Gene Polymorphism and Resistance Training Response Outcomes in Total Participants.

Testing Metric	Genotype Group	Pre (Mean ± SD)	Post (Mean ± SD)	*P*-value (Post Group Diff)
**rs731236 (*TaqI*)**				
**Muscle Strength**				
Back Squat 1RM (kg)	AA (*n*=170)	76.33 ± 31.03	113.09 ± 38.06$^{\blacktriangle\blacktriangle}$	> 0.05
	AG (*n*=21)	87.57 ± 29.04	125.33 ± 33.70$^{\blacktriangle\blacktriangle}$	
Bench Press 1RM (kg)	AA (*n*=170)	39.40 ± 20.04	54.72 ± 22.89$^{\blacktriangle\blacktriangle}$	> 0.05
	AG (*n*=21)	48.10 ± 22.05	62.75 ± 22.87$^{\blacktriangle\blacktriangle}$	
Isokinetic Flexion Total Work (J)	AA (*n*=170)	838.72 ± 368.96	911.16 ± 374.83$^{\blacktriangle\blacktriangle}$	> 0.05
	AG (*n*=21)	930.15 ± 346.72	1003.25 ± 345.01	
Isokinetic Extension Total Work (J)	AA (*n*=170)	878.76 ± 382.65	945.71 ± 392.77$^{\blacktriangle\blacktriangle}$	> 0.05
	AG (*n*=21)	965.05 ± 327.34	1005.60 ± 329.17	
Isometric Leg Press Force (N)	AA (*n*=170)	3171.73 ± 1141.67	3390.48 ± 1291.17$^{\blacktriangle\blacktriangle}$	> 0.05
	AG (*n*=21)	3702.44 ± 1020.54	3913.94 ± 1608.02	
**Power**				
CMJ Relative Peak Power (W/kg)	AA (*n*=170)	21.68 ± 6.68	24.50 ± 6.62$^{\blacktriangle\blacktriangle}$	> 0.05
	AG (*n*=21)	22.57 ± 6.79	24.80 ± 7.76$^{\blacktriangle}$	
**Body Composition**				
Whole-Body Bone Mineral Content (g)	AA (*n*=170)	2678.58 ± 533.61	2708.86 ± 546.26$^{\blacktriangle}$	> 0.05
	AG (*n*=21)	2860.61 ± 461.53	2917.76 ± 509.38	

Testing Metric	Genotype Group	Pre (Mean ± SD)	Post (Mean ± SD)	*P*-value (Post Group Diff)
Upper Limb Bone Mineral Content (g)	AA (*n*=170)	168.60 ± 43.23	163.43 ± 44.65	0.014*
	AG (*n*=21)	181.23 ± 38.95	188.79 ± 46.24	
Body Weight (kg)	AA (*n*=170)	63.78 ± 13.28	64.93 ± 12.93▲	> 0.05
	AG (*n*=21)	67.49 ± 13.33	67.51 ± 12.96	
Whole-Body Muscle Mass (g)	AA (*n*=170)	44020.70 ± 9723.48	46566.78 ± 10335.97▲▲	> 0.05
	AG (*n*=21)	49729.00 ± 9574.56	49729.90 ± 10160.23▲▲	
**Muscle Morphology**				
Pectoralis Major Muscle Thickness (cm)	AA (*n*=170)	1.15 ± 0.38	1.39 ± 0.47▲▲	> 0.05
	AG (*n*=21)	1.31 ± 0.43	1.54 ± 0.59▲▲	
Combined Thickness of Rectus Femoris and Vastus Intermedius Muscles (cm)	AA (*n*=170)	3.86 ± 0.77	4.59 ± 0.76▲▲	> 0.05
	AG (*n*=21)	4.12 ± 0.63	4.35 ± 1.17▲▲	
**rs7975232 (*ApaI*)**				
**Muscle Strength**				
Back Squat 1RM (kg)	AA (*n*=10)	77.34 ± 36.75	115.90 ± 42.94▲▲	> 0.05
	AC (*n*=72)	79.94 ± 28.17	118.51 ± 35.90▲▲	
	CC (*n*=109)	75.90 ± 32.22	111.47 ± 38.35▲▲	
Bench Press 1RM (kg)	AA (*n*=10)	36.26 ± 21.95	51.90 ± 24.03▲▲	> 0.05
	AC (*n*=72)	42.35 ± 19.99	57.91 ± 22.30▲▲	
	CC (*n*=109)	39.36 ± 20.47	54.40 ± 23.27▲▲	

Testing Metric	Genotype Group	Pre (Mean ± SD)	Post (Mean ± SD)	*P*-value (Post Group Diff)
Isokinetic Flexion Total Work (J)	AA (*n*=10)	760.10 ± 289.09	757.70 ± 248.55	> 0.05
	AC (*n*=72)	849.49 ± 363.40	934.13 ± 350.17▲	
	CC (*n*=109)	855.29 ± 378.70	890.30 ± 389.61▲	
Isokinetic Extension Total Work (J)	AA (*n*=10)	775.20 ± 381.63	738.10 ± 224.49	> 0.05
	AC (*n*=72)	898.23 ± 361.99	956.33 ± 342.02▲	
	CC (*n*=109)	928.11 ± 393.34	969.90 ± 420.25▲	
Isometric Leg Press Force (N)	AA (*n*=10)	2567.25 ± 1210.65	3238.13 ± 2056.60	> 0.05
	AC (*n*=72)	3286.85 ± 1042.24	3384.81 ± 1303.48	
	CC (*n*=109)	3230.95 ± 1183.42	3508.38 ± 1292.72▲▲	
**Power**				
CMJ Relative Peak Power (W/kg)	AA (*n*=10)	20.06 ± 5.34	24.03 ± 6.86▲	> 0.05
	AC (*n*=72)	22.42 ± 7.23	25.25 ± 7.47▲▲	
	CC (*n*=109)	21.56 ± 6.39	24.23 ± 6.35▲▲	
**Body Composition**				
Whole-Body Bone Mineral Content (g)	AA (*n*=10)	2494.55 ± 473.64	2479.65 ± 493.00	> 0.05
	AC (*n*=72)	2736.26 ± 580.52	2777.09 ± 484.75	
	CC (*n*=109)	2691.24 ± 560.63	2725.59 ± 582.38▲	
Upper Limb Bone Mineral Content (g)	AA (*n*=10)	156.80 ± 44.53	160.05 ± 46.52	> 0.05
	AC (*n*=72)	168.38 ± 44.39	171.07 ± 48.65▲	

Testing Metric	Genotype Group	Pre (Mean ± SD)	Post (Mean ± SD)	*P*-value (Post Group Diff)
	CC (*n*=109)	162.91 ± 4227	163.58 ± 43.18	
Body Weight (kg)	AA (*n*=10)	58.61 ± 8.22	58.86 ± 7.39	> 0.05
	AC (*n*=72)	64.92 ± 13.66	65.25 ± 12.82	
	CC (*n*=109)	64.19 ± 13.34	65.75 ± 13.25▲▲	
Whole-Body Muscle Mass (g)	AA (*n*=10)	42924.20 ± 8072.56	43922.07 ± 7956.40▲	> 0.05
	AC (*n*=72)	44946.50 ± 9699.43	47647.10 ± 10121.90▲▲	
	CC (*n*=109)	44255.01 ± 9971.46	46756.27 ± 10679.40▲▲	
**Muscle Morphology**				
Pectoralis Major Muscle Thickness (cm)	AA (*n*=10)	1.06 ± 0.42	1.29 ± 0.44	> 0.05
	AC (*n*=72)	1.14 ± 0.34	1.35 ± 0.43▲▲	
	CC (*n*=109)	1.20 ± 0.41	1.39 ± 0.49▲▲	
Combined Thickness of Rectus Femoris and Vastus Intermedius Muscles (cm)	AA (*n*=10)	3.74 ± 0.53	4.25 ± 0.48▲▲	> 0.05
	AC (*n*=72)	3.92 ± 0.69	4.51 ± 0.85▲▲	
	CC (*n*=109)	3.88 ± 0.83	4.63 ± 0.81▲▲	
**rs1544410 (*BsmI*)**				
**Muscle Strength**				
Back Squat 1RM (kg)	CC (*n*=169)	76.40 ± 31.01	113.16 ± 38.06▲▲	> 0.05
	CT (*n*=22)	86.55 ± 29.60	124.27 ± 34.14▲▲	
Bench Press 1RM (kg)	CC (*n*=169)	39.49 ± 20.00	54.83 ± 22.81▲▲	> 0.05

Testing Metric	Genotype Group	Pre (Mean ± SD)	Post (Mean ± SD)	*P*-value (Post Group Diff)
	CT (*n*=22)	46.95 ± 22.60	61.95 ± 23.70▲▲	
Isokinetic Flexion Total Work (J)	CC (*n*=169)	835.08 ± 369.60	915.04 ± 374.80▲▲	> 0.05
	CT (*n*=22)	954.43 ± 333.66	968.52 ± 353.83	
Isokinetic Extension Total Work (J)	CC (*n*=169)	875.65 ± 383.79	949.44 ± 391.93▲▲	> 0.05
	CT (*n*=22)	985.24 ± 312.18	973.81 ± 344.40	
Isometric Leg Press Force (N)	CC (*n*=169)	3185.77 ± 1149.10	3405.08 ± 1290.49▲▲	> 0.05
	CT (*n*=22)	3557.00 ± 1014.91	3764.21 ± 1635.53	
**Power**				
CMJ Relative Peak Power (W/kg)	CC (*n*=169)	21.74 ± 6.66	24.53 ± 6.59▲▲	> 0.05
	CT (*n*=22)	22.14 ± 7.00	24.52 ± 7.92▲▲	
**Body Composition**				
Whole-Body Bone Mineral Content (g)	CC (*n*=169)	2683.31 ± 535.54	2714.09 ± 548.66▲	> 0.05
	CT (*n*=22)	2817.49 ± 461.88	2869.73 ± 507.47	
Upper Limb Bone Mineral Content (g)	CC (*n*=169)	162.75 ± 43.31	163.58 ± 44.76	0.026*
	CT (*n*=22)	179.23 ± 39.15	186.50 ± 46.21	
Body Weight (kg)	CC (*n*=169)	63.86 ± 13.27	65.02 ± 12.91▲▲	> 0.05
	CT (*n*=22)	66.66 ± 13.55	66.72 ± 13.21	
Whole-Body Muscle Mass (g)	CC (*n*=169)	44105.09 ± 9777.26	46663.07 ± 10392.80▲▲	> 0.05
	CT (*n*=22)	46907.58 ± 9458.12	48662.10 ± 9947.23▲▲	

Testing Metric	Genotype Group	Pre (Mean ± SD)	Post (Mean ± SD)	*P*-value (Post Group Diff)
**Muscle Morphology**				
Pectoralis Major Muscle Thickness (cm)	CC (*n*=169)	1.15 ± 0.38	1.39 ± 0.47▲	> 0.05
	CT (*n*=22)	1.29 ± 0.42	1.54 ± 0.59▲	
Combined Thickness of Rectus Femoris and Vastus Intermedius Muscles (cm)	CC (*n*=169)	3.85 ± 0.77	4.59 ± 0.77▲	> 0.05
	CT (*n*=22)	4.11 ± 0.64	4.38 ± 1.14	

Note: ▲ indicates a significant within-group difference pre- vs. post-intervention with $P < 0.05$; ▲▲ indicates $P < 0.01$. * indicates significant differences among different genotype groups post-intervention $P < 0.05$.
